# Custom made inclusion bodies: impact of classical process parameters and physiological parameters on inclusion body quality attributes

**DOI:** 10.1186/s12934-018-0997-5

**Published:** 2018-09-20

**Authors:** Christoph Slouka, Julian Kopp, Stefan Hutwimmer, Michael Strahammer, Daniel Strohmer, Elisabeth Eitenberger, Andreas Schwaighofer, Christoph Herwig

**Affiliations:** 1 Christian Doppler Laboratory for Mechanistic and Physiological Methods for Improved Bioprocesses, Institute of Chemical Engineering, Vienna University of Technology, Vienna, Austria; 2 Sandoz GmbH, Biochemiestrasse 10, 6250 Kundl, Tirol Austria; 3 Institute of Chemical Technology and Analytics, Getreidemarkt 9/164, 1060 Vienna, Austria; 4 Institute of Chemical, Environmental and Bioscience Engineering, Research Area Biochemical Engineering, Vienna University of Technology, Gumpendorfer Strasse 1a, 1060 Vienna, Austria

**Keywords:** *Escherichia coli*, Inclusion body quality attributes, Recombinant protein production, Upstream development

## Abstract

**Background:**

The bacterium *E. coli* is a major host for recombinant protein production of non-glycosylated products. Depending on the expression strategy, the recombinant protein can be located intracellularly. In many cases the formation of inclusion bodies (IBs), protein aggregates inside of the cytoplasm of the cell, is favored in order to achieve high productivities and to cope with toxic products. However, subsequent downstream processing, including homogenization of the cells, centrifugation or solubilization of the IBs, is prone to variable process performance or can be characterized by low extraction yields as published elsewhere. It is hypothesized that variations in IB quality attributes (QA) are responsible for those effects and that such attributes can be controlled by upstream process conditions. This contribution is aimed at analyzing how standard process parameters, such as pH and temperature (T) as well as different controlled levels of physiological parameters, such as specific substrate uptake rates, can vary IB quality attributes.

**Results:**

Classical process parameters like pH and T influence the expression of analyzed IB. The effect on the three QAs titer, size and purity could be successfully revealed. The developed data driven model showed that low temperatures and low pH are favorable for the expression of the two tested industrially relevant proteins. Based on this knowledge, physiological control using specific substrate feeding rate (of glucose) q_s,Glu_ is altered and the impact is tested for one protein.

**Conclusions:**

Time dependent monitoring of IB QA—titer, purity, IB bead size—showed a dependence on classical process parameters pH and temperature. These findings are confirmed using a second industrially relevant strain. Optimized process conditions for pH and temperature were used to determine dependence on the physiological parameters, the specific substrate uptake rate (q_s,Glu_). Higher q_s,Glu_ were shown to have a strong influence on the analyzed IB QAs and drastically increase the titer and purity in early time stages. We therefore present a novel approach to modulate—time dependently—quality attributes in upstream processing to enable robust downstream processing.

**Electronic supplementary material:**

The online version of this article (10.1186/s12934-018-0997-5) contains supplementary material, which is available to authorized users.

## Background

The gram-negative bacterium *E. coli* is the expression host of choice for the production of 30–40% of recombinant drugs in industry [1, 2]. As *E. coli* shows very fast replication rates [3, 4] on comparatively inexpensive media [5], the benefits often outweigh the numerous purification steps [1, 6] and the missing glycosylation pattern [1, 7, 8]. Recombinant protein production in *E. coli* regained more interest as the demand in single chain antibody-fragments increased, which can be properly expressed in *E. coli* [1, 8]. The strain BL21(DE3) created by F. Studier and B. Moffatt back in 1986 [9] is often used in an industrial scale, because of very low acetate formation, high replication rates [9–14], as well as the possibility of protein secretion into the fermentation broth due to a type 2 secretion protein [15–17]. For expression of the recombinant protein, the lac operon is still one of the most favored promotors in pET-expression-systems using integrated T7-polymerase for high transcriptional rates [3, 12, 18]. The repressor protein can only be blocked by allolactose or a structural analogue [19], e.g. the well-known expensive inducer isopropyl β-d-1 thiogalactopyranoside (IPTG) [3, 13]. However, induction with IPTG stresses the cells, as IPTG in higher concentrations is known to be toxic [13, 18, 20].

Recombinant proteins are often expressed as inclusion bodies (IB). IBs have originally been believed to be waste products by bacteria [21], until it was realized that they are formed as a stress reaction by the cells resulting in a biologically inactive precipitated protein [22–24]. Such stress reactions can be caused by high temperatures, pH-shifts or occur due to high feeding rates. These factors tend to result in higher yields of product [1], which of course are advantageous combined with the possibility of expressing toxic proteins [6]. Still, the DSP and especially the refolding unit operation suffers in robustness and is the most time-consuming step in gaining the correctly folded product from *E. coli* cultivations [21–24], which requires significantly more technology and time, when purifying IBs [22, 25, 26].

Quality attributes (or key performance indicator) of IBs, such as titer and morphology changes during extraction procedures have already been studied and show that IBs are dynamic structures depending on the cultivation and extraction conditions [27–29]. First approaches towards IB sizing in the upstream process have already been made within our group by Reichelt et al. [30] using transmission electron microscopy [31] in combination with nanoparticle tracking analysis (NTA) revealing general trends of IB growth during cultivation. Further studies show that IBs consist of up to 50% correctly folded protein in contrast to the general perception of IBs as inactive structures [29, 32]. Combined with the fact that IBs can be produced in high concentration (so that the amount of generated product often outweighs the additional downstream steps), IB based processes are believed to fundamentally boost time/space yields for recombinant protein production [1, 6, 7, 21]. Knowledge about the state of IB QAs during a cultivation process is therefore of utmost importance. Three IB QAs are generally of importance: bead size, titer and purity, as those three quality attributes were already defined elsewhere [21, 30, 33, 34]. It has been reported that inclusion body sizes can be measured with different methods, e.g. AFM (atomic force microscopy), TEM and NTA [21, 30, 33]. SDS-pages and ELISA-methods have been often reported as tool to determine impurities and titer in the IB product samples [35]. The impact of single process parameters like pH on IB QAs has already been investigated in literature [36, 37]. Reichelt et al. [34] showed that alterations of (q_s,glu_) influence the behavior of common IB-processes, using IPTG as an inducer. The impact of the feeding rate onto product formation in *E. coli* BL21(DE3) has been investigated recently, though lactose was used as inducer instead of IPTG [38]. However, no monitoring of all IB-QAs over induction time has been performed in any of the previous studies.

In this study we performed cultivations with a BL21(DE3) strain, producing a recombinant protein coupled to a N-pro-fusion protein [39]—*strain 1*—and a non N-Pro fused protein—*strain 2*-, both exclusively expressing IBs, as the products are highly toxic for the cell. Classical process parameters were monitored as a function of induction time. The impact of process parameters on IB bead size in combination with purity and titer as a function of time has not been investigated in depth. Secondary structure of different IB sizes were analyzed using IR and showed no differences for IB beads of different size compared to the standard. Based on these results, the physiological parameter of the specific substrate uptake rate (q_s,glu_) is altered at constant pH and T for *strain 1* and QAs are analyzed time-dependently. In this current study we collected time resolved results, which are used to optimize the USP. In conclusion, it is demonstrated that low T and low pH in combination with high q_s,glu_ are beneficial for increasing the productivity and robustness of IB based processes for the two tested proteins.

## Methods

### Strains

*Strain 1* was an *E. coli* BL21(DE3) with the pET[30a] plasmid system (kanamycin resistance) for recombinant protein production. The target protein was linked to a N-pro fusion protein used for purification [39]. *Strain 2*, *E. coli* BL21(DE3), (kanamycin resistance) was used for testing the results obtained with *strain 1.* Expression of the protein occurs only as IB since the product is toxic to the cell. No N-Pro tag is fused to this product.

### Bioreactor cultivations

#### Strain 1

All bioreactor and preculture cultivations for *strain 1* were carried out using a defined minimal medium referred to DeLisa et al. [5]. Batch media and the preculture media had the same composition with different amounts of glucose respectively. The glucose concentrations for the phases were: 8 g/L for the preculture, 20 g/L for the batch phase. The feed for fed-batch and induction had a concentration of 300 g/L glucose.

Antibiotic was added throughout all fermentations, resulting in a final concentration of 0.02 g/L of kanamycin. All precultures were performed using 500 mL high yield flasks. They were inoculated with 1.5 mL of bacteria solution stored in cryos at − 80 °C and subsequently cultivated for 20 h at 230 rpm in an Infors HR Multitron shaker (Infors, Bottmingen Switzerland) at 37 °C.

All cultivations were either performed in a stainless-steel Sartorius Biostat Cplus bioreactor (Sartorius, Göttingen, Germany) with 10 L working volume or in a DASGIP Mini bioreactor-4-parallel fermenter system (max. working volume: 2.5 L; Eppendorf, Hamburg, Germany). Cultivation off gas was analyzed by gas sensors—IR for CO_2_ and ZrO_2_ based for O_2_ (Blue Sens Gas analytics, Herten, Germany).

Process control was established using the PIMS Lucullus and the DAS-GIP-control system, DASware-control, which logged the process parameters. During batch-phase and fedbatch phase pH was kept constant at 7.2 and controlled with base only (12.5% NH_4_OH), while acid (5% H_3_PO_4_) was added manually, when necessary. The pH was monitored using an EasyFerm Plus pH-sensor (Hamilton, Reno, NV, USA). The reactors were continuously stirred at 1400 rpm and aerated using a mixture of pressurized air and pure oxygen at 2 vvm. Dissolved oxygen (dO_2_) was always kept higher than 30% by increasing the ratio of oxygen in the ingas. The dissolved oxygen was monitored using a fluorescence dissolved oxygen electrode Visiferm DO (Hamilton, Reno, NV, USA). The fed-batch phase for biomass generation was followed by an induction phase using a feed medium with glucose as primary carbon source.

**Fig. 1:**
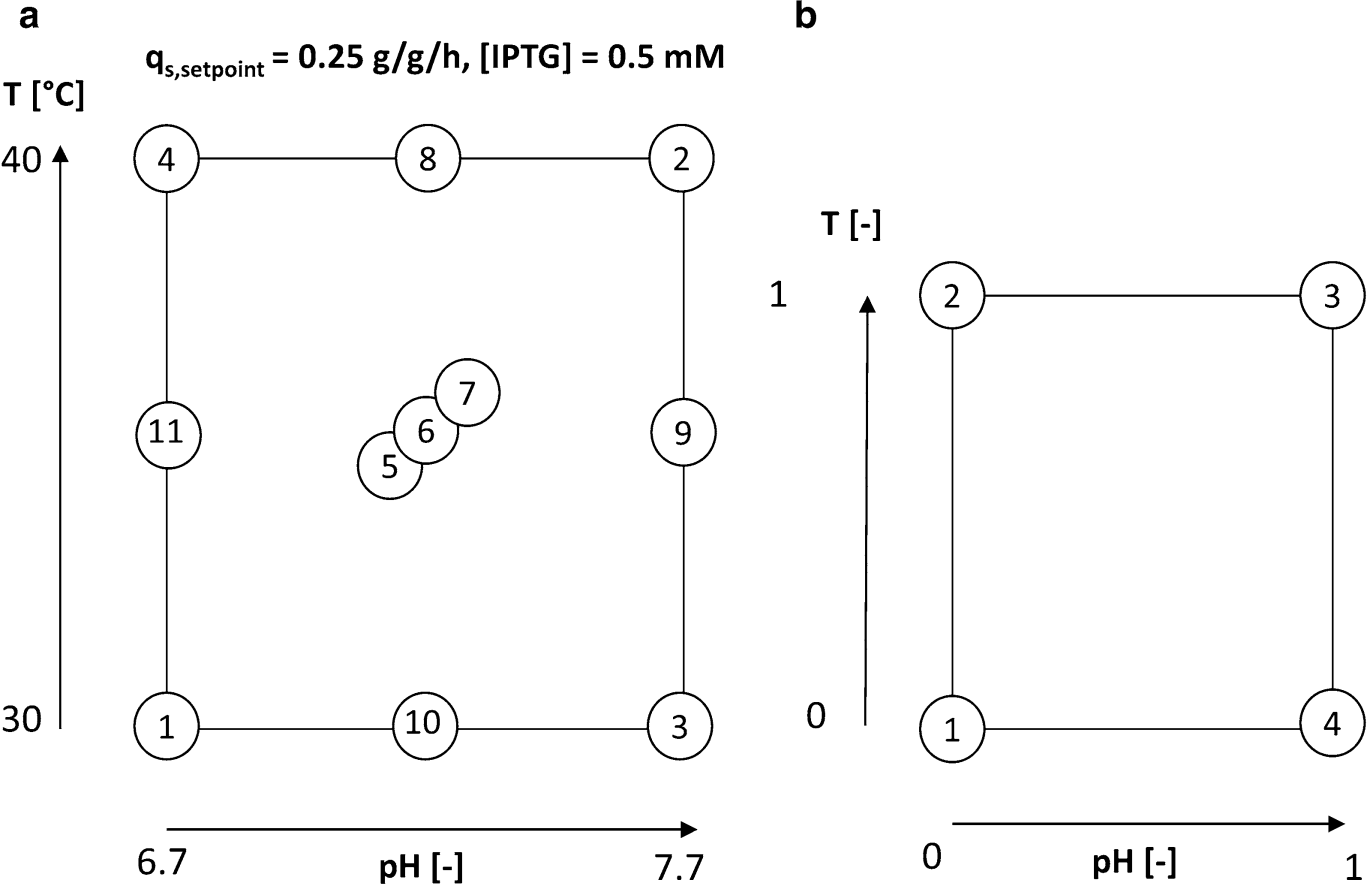
**a** DoE for determination of the influence of classical process parameters on IB QAs for *strain 1*. Starpoints (8, 9, 10, 11) were performed in a DasGip parallel system, while the others were cultivated in a stainless-steel Sartorius Stedim reactor; **b** reduced design space for *strain 2* based on optimal cultivation parameters

0.5 mM IPTG was added as an inducer once to start the induction of the cells. pH and temperature in the induction phase was adapted according to the design of experiments (DoE) given in Fig. 1a. pH was altered between 6.7 and 7.7 and temperature between 30 and 40 °C. The center point at 35 °C and pH 7.2 was cultivated in triplicate in order to assess statistical experimental error.

#### Strain 2

*Strain 2* was cultivated at our industrial partner. The cultivation was similar to *strain 1* using chemically defined medium containing 15 g/L glucose in seed and 10 g/L glucose in main stage fermentations, respectively. Inoculum preparation and respective antibiotic selection were similar to *strain 1*, though during the main culture stage kanamycin was added. Seed and main culture cultivations were carried out in custom built 50 L stainless steel vessels with custom made fermentation software for process control. Throughout the seed and main fermentation stages the pH was adjusted to fit the parameters of the second DoE (Fig. 1b) using 150 g/L sulphuric acid or 25% ammonia. Temperature was adjusted to the corresponding values in main culture. Dissolved oxygen was adjusted to 30% using aeration with up to 2 vvm, 2 bar backpressure and stirring up to 500 rpm. Optical DO probes Visipro DO (Hamilton, Reno, NV, USA) and EasyFerm Plus pH probes (Mettler Toledo, Columbus, Ohio; USA) were used for monitoring and control. Off-gas analysis was conducted using a custom-built mass spectrometer facility. At OD_600_ > 8.5 in seed culture, main culture was inoculated using 8.6% (v/v). Upon glucose depletion a glucose feed was initiated using a µ of 0.3 h^−1^ for 6 h and was kept constant at a final rate of exponential feed pattern until process termination. Expression was induced 2 h after the end of exponential feeding for biomass production using 1 mM IPTG for 12 h in a reduced design space given in Fig. 1b. As high temperatures and alkaline pH (fermentation conditions 2 in Fig. 1a) showed pronounced lysis during the study, the design space for strain 2 was reduced to a more reasonable pH and temperature window which is commonly used for multiple *E. coli* cultivations. Absolute values for pH and T cannot be given due to confidential reasons by our industrial partner.

### Cultivation analytics

#### Biomass

For dry cell weight (DCW) measurements 1 mL of the cultivation broth was centrifuged at 9000 rpm, subsequently washed with 0.9% NaCl solution and centrifuged again under the same conditions. After drying the cells at 105 °C for 48 h the pellet was evaluated gravimetrically. DCW measurements were performed in five replicates and the mean error for DCW was about 3%. Offline OD_600_ measurements were performed in duplicates in a UV/VIS photometer Genisys 20 (Thermo Scientific, Waltham, MA, US).

#### Flow cytometry

Flow cytometry (FCM) was carried out according to Langemann et al. [36]. We used a CyFlow^®^ Cube 6 flow cytometer (Partec, Münster, Germany) with 488-nm blue solid-state lasers. Three fluorescence channels were available (FL1, 536/40 nm bandpass; FL2, 570/50 nm bandpass; FL3, 675 nm longpass) alongside forward scatter (trigger parameter) and side scatter detection. This device featured true absolute volumetric counting with a sample size of 50–100 μL. Data were collected using the software CyView 13 (Cube 6; Partec) and analyzed with the software FCS Express V.4.07.0001 (DeNovo Software, Los Angeles, CA, USA). Membrane potential-sensitive dye DiBAC_4_(3) (abs./em. 493/516 nm) was used for the assessment of viability. Fluorescent dye RH414 (abs./em. 532/760 nm) was used for staining of plasma membranes yielding strong red fluorescent enhancement for the analysis of total cell number. Combining those two dyes it was possible to quantify the viable cell concentration. Stocks of 0.5 mM (DiBAC_4_(3)) and 2 mM RH414 were prepared in dimethyl sulfoxide and stored at − 20 °C. Both dyes were purchased from AnaSpec (Fremont CA, USA). 1.5 μL of both stocks were added to 1 mL diluted sample resulting in final concentrations of 0.5 μM DiBAC_4_(3) and 2.0 μM RH414, respectively. Samples were measured directly after addition of the dyes, without further incubation.

#### Sugar analytics

Sugar concentrations in the filtered fermentation broth were determined using a Supelco C-610H HPLC column (Supelco, Bellefonte, PA, USA) on an Ultimate 300 HPLC system (Thermo Scientific, Waltham, MA, US) using 0.1% H_3_PO_4_ as running buffer at 0.5 mL/min or an Aminex HPLC column (Biorad, Hercules; CA, USA) on an Agilent 1100 System (Agilent Systems, Santa Clara, CA, USA) with 4 mM H_2_SO_4_ as running buffer at 0.6 mL/min.

### Product analytics

#### IB preparation

5 mL fermentation broth samples were centrifuged at 4800 rpm at 4 °C. The supernatant is discarded and the pellet is resuspended to a DCW of about 4 g/L in lysis buffer (100 mM Tris, 10 mM EDTA at pH 7.4). Afterwards the sample was homogenized using a high-pressure homogenizer at 1500 bar for 10 passages (Emulsiflex C3; Avestin, Ottawa, Canada). After centrifugation at 10,000 rpm and 4 °C the supernatant was discarded and the resulting IB pellet was washed twice with ultrapure water and aliquoted into pellets à 2 mL broth, centrifuged (14,000 rpm, 10 min 4 °C) and stored at − 20 °C.

#### IB size

**Fig. 2:**
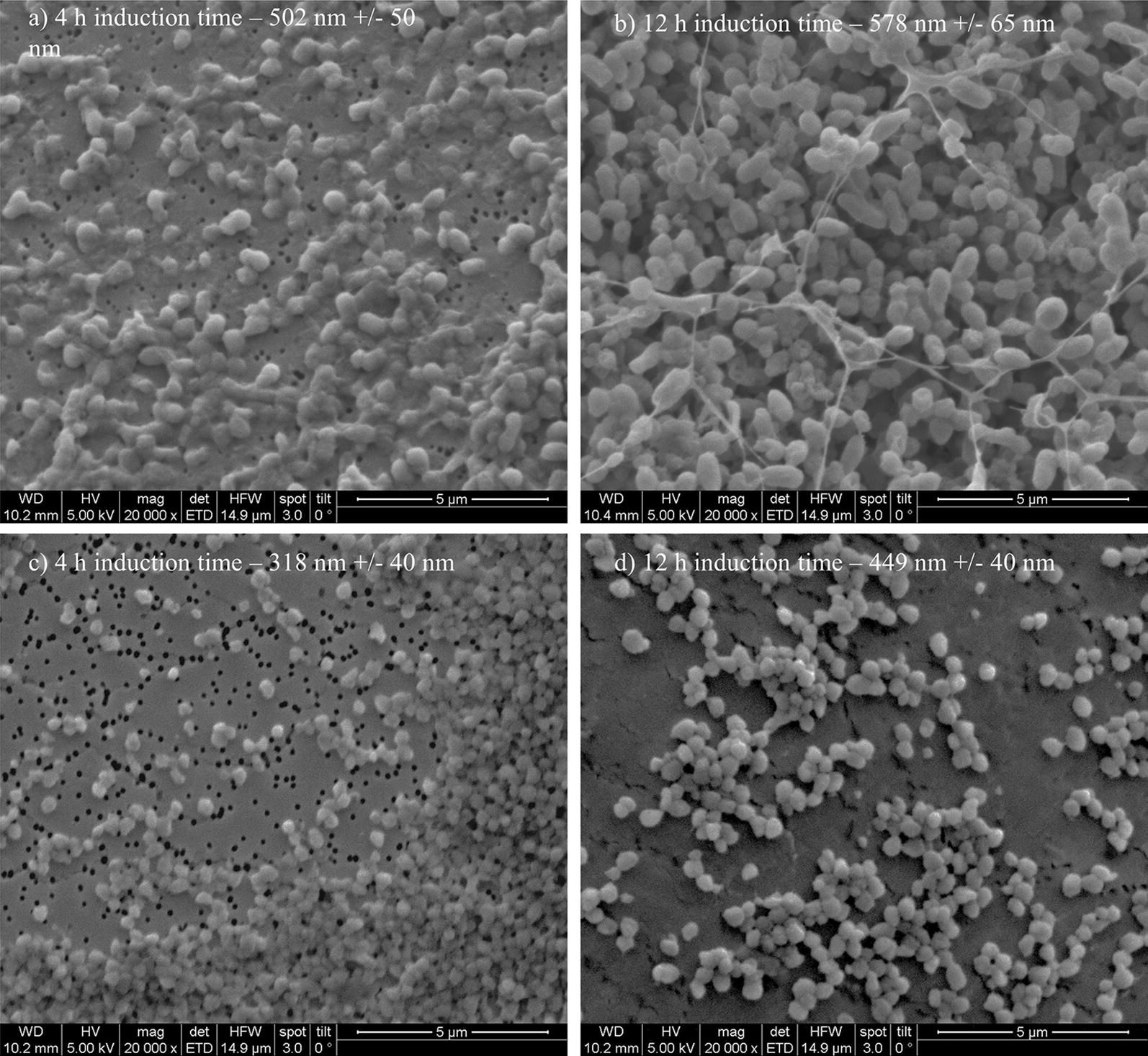
Extracted IBs filtered onto Au coated polycarbonate filter and analyzed using SEM for 4 h induction time and 12 h induction time. Strong difference in size can be spotted for the two-time points

Washed and aliquoted IB samples were resuspended in ultrapure water. 100 µL of appropriate dilution of the suspension were pipetted on a gold-sputtered (10–50 nm) polycarbonate filter (Millipore-Merck, Darmstadt, Germany) using reusable syringe filter holders with a diameter of 13 mm (Sartorius, Göttingen, Germany). 100 µL of ultrapure water were added and pressurized air was used for subsequent filtration. Additional 200 µL of ultrapure water were used for washing. The wet filters were fixed on a SEM sample holder using graphite adhesive tape and subsequently sputtered with gold to increase the contrast of the sample. SEM was performed using a QUANTA FEI SEM (Thermo Fisher, Waltham, MA, US) with a secondary electron detector [40]. The acceleration voltage of the electron beam was set between 3 and 5 kV. To determine the diameter of the IBs, 50 IBs on SEM pictures were measured using the ImageJ plugin Fiji [Laboratory for Optical and Computational Instrumentation (LOCI), University of Wisconsin-Madison, US]. SEM analytics of two different time points for both strains are given in Fig. 2.

#### IB titer for strain 1

For titer measurements IB pellets were solubilized using solubilization buffer (7.5 M guanidine hydrochloride, 62 mM Tris at pH 8). The filtered samples are quantified by HPLC analysis (UltiMate 3000; Thermo Fisher, Waltham, MA, USA) using a reversed phase column (EC 150/4.6 Nucleosil 300-5 C8; Macherey–Nagel, Düren, Germany). The product was quantified with an UV detector (Thermo Fisher, Waltham, MA, USA) at 214 nm using Novartis BVS Ref. 02 as standard. Mobile phase was composed of acetonitrile and water both supplemented with 0.1% (v/v) trifluoride acetic acid. A linear gradient from 30% (v/v) acetonitrile to 100% acetonitrile (ACN) was applied. A steep linear gradient from 10% ACN to 30% ACN in 60 s was followed by a long linear gradient from 30 to 55% and by three regeneration steps.

#### IB titer for  strain 2

IB titer was also determined by reversed phase HPLC at Sandoz GmbH (Process Analytics, Kundl, Tirol, Austria). Pellets were defrosted at room temperature and solubilized by addition of dilution buffer [36] (6 M guanidine hydrochloride, 50 mM Tris, pH 7.5) and sonication (Branson Ultrasonics, Danbury, Connecticut, USA). The filtered samples were analyzed by HPLC with a reversed phase column (Acquity UPLC BEH 300, C4, 1.7 µm, 2.1 × 50 mm). Quantification was performed by UV detection at 214 nm wavelength and calibration with a purified product standard. Mobile phases were composed of (A) water and (B) acetonitrile/pentanol (95/5, v/v) both supplemented with 0.1% (v/v) tetrafluoride acetic acid. The elution of the product was achieved with a linear gradient of both solvents.

#### IB purity

Purity measurements were performed using chip-based protein assays with 2100 Bioanalyzer (Agilent Technologies, Santa Clara, CA, USA. The chip-based assay is based on SDS-PAGE and therefore separates molecules according to their size. Washed and homogenized IBs were dissolved in 3 M urea, 25 mM Tris at pH 7 and measured subsequently. The electropherogram was afterwards analyzed using OriginPro 2016 (Northampton, MA, USA) integrating the peak area of the protein of interest and normalizing the area in respect to the total area of the electropherogram.

#### IB conformational analysis by IR spectroscopy

Infrared (IR) spectra were recorded by an external-cavity quantum cascade laser-based IR transmission setup described in detail by Schwaighofer et al. [31]. A water-cooled external-cavity quantum cascade laser (Hedgehog, Daylight Solutions Inc., San Diego, USA) was used operating at a repetition rate of 100 kHz and a pulse width of 5000 ns. All spectra were recorded in the spectral tuning range between 1730 and 1470 cm^−1^, covering the amide I and amide II region of proteins, at a scan speed of 1200 cm^−1^ s^−1^. The MIR light was focused on the detector element by a gold plated off-axis parabolic mirror with a focal length of 43 mm. A thermoelectrically-cooled MCT detector operating at − 78 °C (PCI-10.6, Vigo Systems S.A., Poland) was used as IR detector. To reduce the influence of water vapor, the setup was placed in a housing of polyethylene foil and constantly flushed with dry air. The measured signal was processed by a lock-in amplifier (Stanford Research Systems, CA, USA) and digitized by a NI DAQ 9239 24-bit ADC (National Instruments Corp., Austin, USA). Each single beam spectrum consisting of 6000 data points was recorded during the tuning time for one scan of approx. 250 µs. A total of 100 scans were recorded for background and sample single beam spectra at a total acquisition time of 53 s. All measurements were carried out using a custom-built, temperature-controlled flow cell equipped with two MIR transparent CaF_2_ windows and 31 µm-thick spacer, at 20 °C.

The laser was controlled by Daylight Solution driver software; data acquisition and temperature control were performed using a custom-made LabView-based GUI (National Instruments Corp., Austin, USA). Two IB samples with distinct size of 400 nm and 600 nm were compared with the finished formulated protein standard of *strain 1* (without N-Pro Taq).

## Results and discussion

The goal of this study was to investigate and to understand if and how IB attributes can be changed and tuned by upstream bioprocess (USP) technological methods. We tested the classical process parameters pH and temperature and the physiological parameter specific substrate uptake rate. The impact of specific USP parameters can be investigated using IB QAs as response for data evaluation. With knowledge about the tunability of IB QAs in the upstream, it is possible to simplify the subsequent downstream steps. Therefore, we tested two different proteins, with completely different structure including N-Pro fusion tag for *strain 1* and no fusion tag for *strain 2.* Both products have a high toxicity for the cell in common and are only expressed as IBs. The results constitute the key to custom made IBs and may be used as platform technology for the development of the USP for new products.

### Impact of classical process parameters on IB QAs using *strain 1* (N-Pro fused protein)

**Fig. 3:**
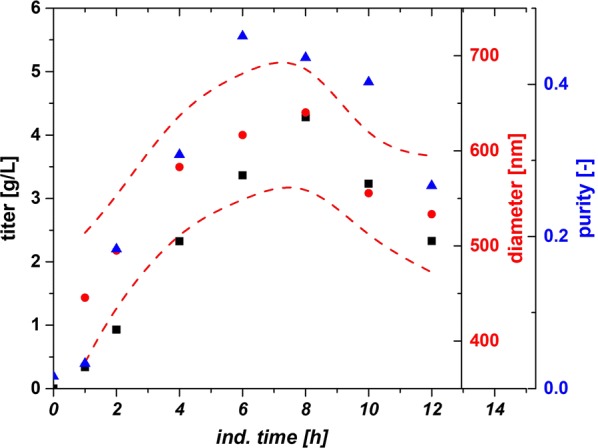
IB QAs as a function of induction time for the third center point cultivation. Size is given with standard deviation (spline). Drop of titer/size and purity after 8 h is generally a result of increased cell lysis at elevated times

**Fig. 4:**
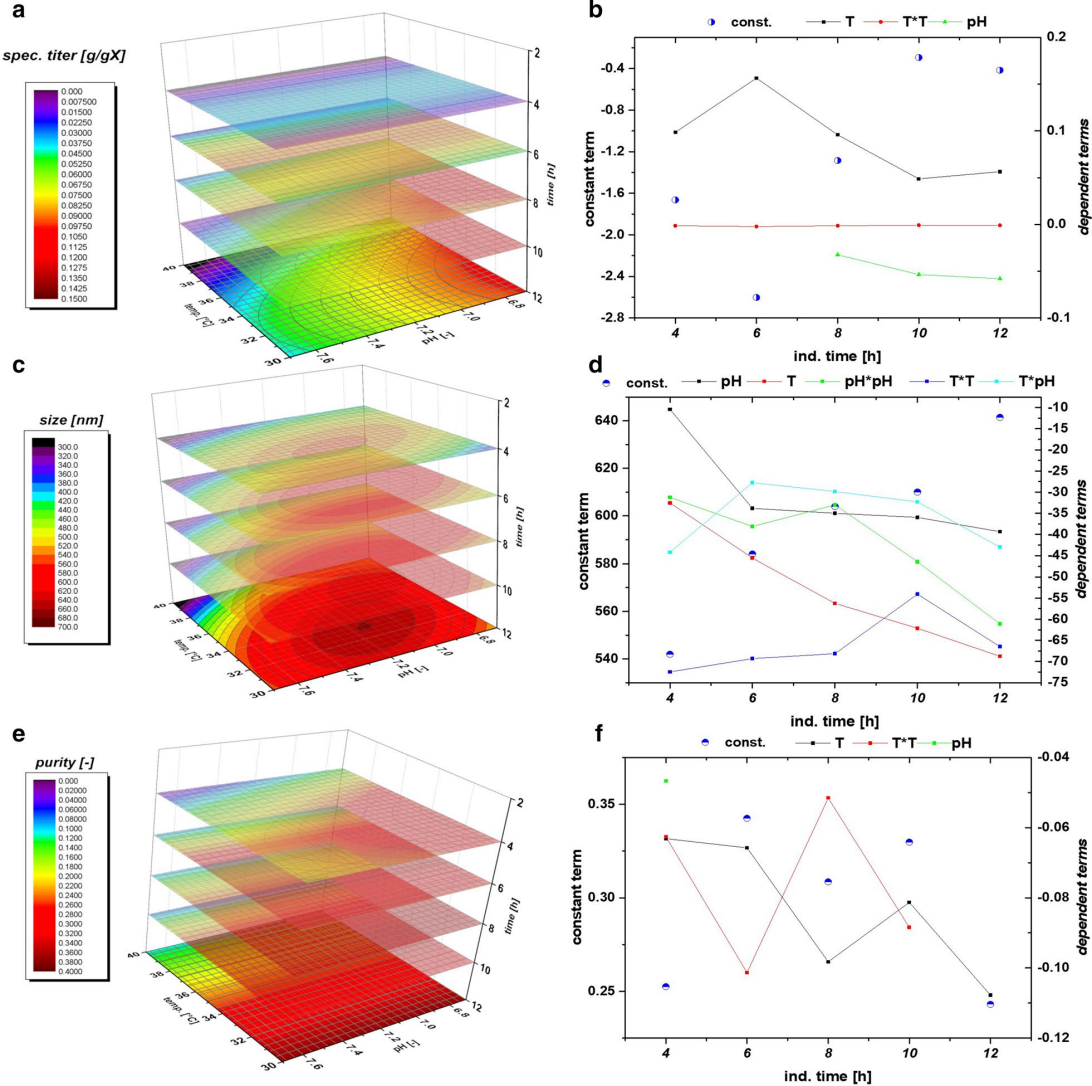
**a** Data driven model for time dependent analysis of IB specific titer; **b** model fit parameter for titer. While in the beginning only temperature dependence is visible, a strong pH correlation can be found at t = 8 h; **c** data driven model for time dependent analysis of IB bead size; **d** model fit parameter for IB bead size. Due to standard deviation of 10% in the analysis model parameters are rather complex; **e** data driven model for time dependent analysis of IB purity; **f** model fit parameter for purity. A sole temperature dependence is found beyond 4 h of induction

As IPTG based induction imposes a metabolic stress to the host organism, time dependent analysis of IB QAs is of utmost importance to identify critical process time points (e.g. cell death, product degradation) within individual cultivation runs. Therefore, IB QAs were analyzed every 2 h within a maximum of 12 h induction time. pH and T were altered based on the experimental plan, while specific substrate uptake rate (q_s,Glu_) and inducer concentration were kept constant in all experiments. In Table 1 the applied parameters for T, pH and q_s,Glu_ for all performed cultivations in the DoE are displayed. Figure 3 exemplarily shows IB QAs of one single cultivation run as a function of time. The received QAs purity, titer and size are used to build a data driven model using MODDE 10 (Umetrics, Sweden). A partial least square fit was used for all models. Model terms (linear, quadratic and interaction terms) were evaluated according to their validity (p-values) and to the overall model quality. A clear dependence for the applied variations in pH and T were found and visualized against induction time giving a time dependent analysis of the QAs. The evaluation of the specific titer [based on titer (g/L) divided by the biomass at the given timepoint (gX/L, resulting in g/g)] against the induction time and pH and T showed a clear dependence. The specific titer was used in order to compensate for deviations in the biomass after the non-induced fed batch, which yielded 25–30 g/L DCW. The maximum of spec. titer (not necessarily the spec. productivity at certain time point) was found at low T and low pH, shown in Fig. 4a. pH dependence got significant after 6 h of induction time and impacted (Fig. 4b) the spec. titer. The maximum of recombinant protein was produced between 8 and 10 h. This fact is well reflected by the const. parameter in Fig. 4b. After 10 h cell death leads to a degradation and reduction of the produced protein, also clearly deducible from the constant term, visible in FCM measurements and in pronounced glucose accumulation (data not shown).

**Table 1:** Analysis of applied process parameters compared to set points in all DoE runs during induction phase

DoE	pH_set_ (−)	T_set_ (°C)	pH_real_ (−)	T_real_ (°C)	q_s,Glu set_ (g/g/h)	q_s,Glu_ _real_ (g/g/h)
1	6.7	30.00	6.69	30.00	0.25	0.24
10	7.2	30.00	7.16	30.02	0.25	0.26
3	7.7	30.00	7.69	30.00	0.25	0.26
11	6.7	35.00	6.64	35.02	0.25	0.29
5	7.2	35.00	7.18	35.00	0.25	0.24
6	7.2	35.00	7.18	35.00	0.25	0.32
9	7.7	35.00	7.64	35.03	0.25	0.27
4	6.7	40.00	6.68	40.00	0.25	0.29
8	7.2	40.00	7.15	40.01	0.25	0.29
2	7.7	40.00	7.69	40.00	0.25	0.24
7	7.2	35.00	7.17	35.00	0.25	0.25

Within single cultivation runs titer and IB bead size showed a very linear relationship in the mean diameter and the standard deviation until the onset of cell death. Process parameters pH and T affected the growth of IB beads significantly. Generally, the largest IB bead size could be found close to the center point of the DoE in the beginning of induction. The shift to lower T and pH can be spotted after 6 h of induction time (compare to Fig. 4c). Effects of cell death and product degradation in titer could also be spotted in the IB bead size especially at 12 h. General trends of the fitting parameters are visualized in Fig. 4d. The constant model parameter is increased over time which also indicates the growth of IB beads over induction time. Linear terms pH and T and quadratic pH term showed increased impact on the model with elevated time, while interaction term and quadratic T-term stayed rather constant. A similar behavior for IB bead growth had already been obtained for a recombinant produced green fluorescent protein (GFP) in our group by Wurm et al. [33]. Instead of altering pH and T like in this study, the induction strength using mixed feed systems with lactose as inducer was varied. Induction time and strength had a high impact on the IB bead size during these cultivations. In our model a certain deregulation of size compared to titer could be dedicated from the given data driven models. This fact is beneficial for regulation of individual parameters to increase the performance in the DSP process chain in a further aspect since size and titer can be varied separately to a certain extend. As third QA IB purity, as important factor for quality in the DSP, was analyzed.

The three-dimensional plot for purity determination is presented in Fig. 4e. At times, up to 4 h of induction pH influenced the purity of the IB samples. After 4 h, a sole dependence on temperature was found indicating that low temperatures (30 °C in the design) favor cleaner IBs. Since titer and size maximum could be found at low temperatures and pH, purity after homogenization may be highly correlated to the degree of lysis during the fermentation run. Lower temperatures did not lead to significant cell death (when regarding up to 10 h of induction), impurities may be reduced by applying low temperatures compared to temperatures with increased cell death yields. So, Fig. 4f summarizes the model fit parameters as a function of time. pH did not contribute to the model fit beyond 4 h (only one point given). Temperature has a major influence on the duration of the induction time, which can already be detected in early stages of induction time. As purity is affected by the washing steps after homogenization different washing procedures may impact the value of absolute purity and the kind of impurity. Generally, porin structures and phospholipids from the outer membrane are the major part of impurities in the IB after homogenization [41, 42]. In literature IB beads had already been analyzed by SEM and AFM in order to get insight into morphology [43] and into washing procedures and dependence of pH and T within [44]. Different washing procedure had also been analyzed in this work. Buffer based washing tends to show little influence in shape and morphology of IBs but has an effect on the analyzed purity value (Additional file 1: Figure S1). This may be attributed to phospholipid content, resulting from homogenization of the cells, as buffer treatment successfully increases purity. Effects of washing on phospholipid content is also reported in [45]. Generally, SDS-PAGE techniques are used to separate different protein sizes. A few impurity peaks are found near the respective fusion protein size of 28.8 kDa and about 60 kDa (Additional file 2: Figure S2 an IB purity for 4 h and 12 h of the validation run). These impurities correlating well to the size-range of a magnitude of outer membrane (e.g. ompA with 35.1 kDA [46]). To determine the extent of DNA in IB as impurities, we treated solubilized IB samples prior to an SDS-PAGE with DNAse 30 min at 37 °C (DNAse 1, Thermo Scientific, Waltham, MA, US). No differences in the gel could be spotted between untreated and treated samples (Additional file 2: Figure S2b). Therefore, we suppose little content of residual DNA within the IB samples, which was also described in [45]. A higher IB purity is based on our model generally attributed to larger IB sizes. Since volume/surface ratio differs drastically compared to small beads less host cell structures can attach to the surface after homogenization. Buffer washing successfully removes a higher content of these impurities.

To evaluate the three data driven model approaches, we performed a verification run, aiming to achieve a maximum in titer of the recombinant protein including prediction of the respective attributes size and purity. Since the maximum of the titer could be found after 10 h of induction time, optimization is performed for this time stage. The process parameters received from the optimization algorithm for the induction phase were pH 6.7, T = 31.5 °C. Table 2 shows the comparison of the model prediction vs. the real measured values received after 10 h of induction. Standard cultivation reproducibility based on center point cultivations of *strain 1* are strongly time dependent, especially for titer and purity assessment. Differences in the real q_s,Glu_ during these three runs may affect the reproducibility, especially in the beginning of the cultivations as will be shown in the forthcoming chapter. Mean values and deviations for the center point runs of *strain 1* are given in Additional file 3: Figure S3. The standard deviation for size is below 10% until 10 h of induction, heading to about 15% at 12 h. Purity shows an error of about 30% for until 8 h reducing to values below 20% afterwards. Low titer values are generally highly defective at early time stages of the induction phase as a result of the onset of production. These high errors of about 30% reduce to about 10% after 8 h of induction. Using these assumptions for evaluation of the model clearly shown that model assumptions for size and purity QAs are correct within the given standard deviations. The IB bead size range after 10 h is predicted correctly, despite the general uncertainty of about 10% in the measurement statistics. Purity was correct within the 20% deviation at this time stage. Even slightly better results could be obtained for titer but are off the 10% deviation. This may be based on the slight higher q_s, Glu_ of 0.3 g/g/h applied in this cultivation (overestimation of biomass after the fed-batch phase). Production of the protein of interest and the expression rate seems to be strongly correlated to the induction stress level of the cell. Lower temperatures seem to be favorable for the survival of the *E. coli* cells and positively influenced all three analyzed quality attributes. pH shifts to low pH increased the titer to a high degree at later induction stages and may be a result of a higher transmembrane potential, boosting the TCA and the energy metabolism [47]. As *E. coli* can grow on a pH between 6.0 and 8.0, with an internal pH of 7.6 [48], the rather acid pH-optimum is surprising at a first glance, but when investigated it is likely that the pH of 6.7 could be causing less precipitate of diverse trace elements, which are added in the DeLisa media [5]. Having access to more co-factors could positively influence the IB-formation. pH shifts from 7.2 to 6.7 may also effect different enzymes in the cell, e.g. phosphofructokinase in glycolysis [48].

**Table 2:** Prediction vs. measured QA of IBs for model validation run

Val. run	Model	Measured	Assessment
Purity	0.345	0.397	Correct within 20% error for purity at 10 h
Spez. titer (optimized)	0.113	0.140	Higher than predicted
Size	570.53	571.63	Prediction correct

### Secondary structure analysis of IBs exhibiting different size

**Fig. 5:**
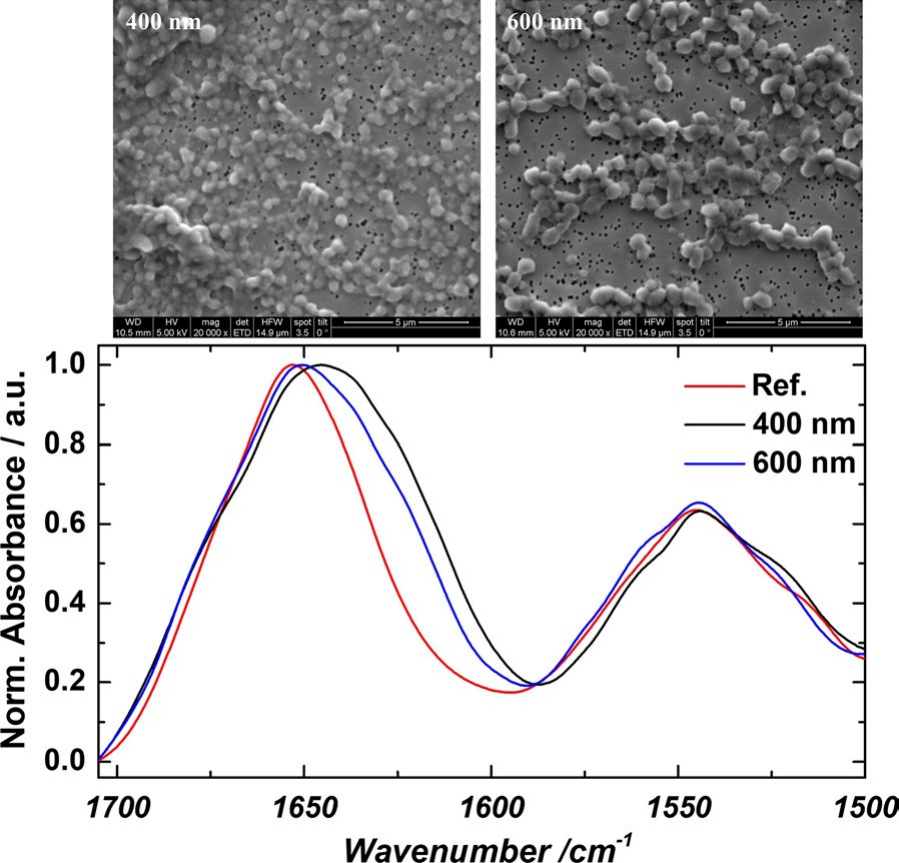
IR spectra of the two distinct bead sizes of 400 and 600 nm, compared to the correctly folded reference sample. SEM analytics of both samples are given above

In order to understand the impact of different IB size (produced in USP) on the secondary structure, IR measurements in the MIR range were performed and compared to the correctly folded protein standard of *strain 1* for two distinct sizes exemplarily. Figure 5 shows the IR spectra of the reference sample and IB beads with 400 and 600 nm in size from the same cultivation run. The IB samples were resuspended in MQ water and subsequently measured. The reference standard was measured in the formulated buffer. The IR spectrum of the reference shows a band maximum at 1645 cm^−1^ in the amide I region as well as a narrow band at 1545 cm^−1^ in the amide II region that are characteristic for α-helical structures. In the reference sample, the native secondary structure of the protein is fully formed. Throughout the fermentation process, 400 nm size sample was taken after 4 h and the 600 nm sample was taken after 8 h. These samples also predominately feature α-helical secondary structure indicated by the amide I band maximum close to 1650 cm^−1^ [49]. However, these samples also contain different, non-native secondary structure as denoted by the band shoulders at approx. 1625 and 1680 cm^−1^ that suggest β-sheet secondary structures. The IR spectra show that the extent of these non-native secondary structure components is different for the two samples taken from the cultivation and that the amount is lower in the sample that was taken at a later point in time. This is in accordance with the purity measurements and indicates that later cultivation times and larger IB sizes do not affect the secondary structure of the IBs negatively. These results can be compared to the work of Wurm et al. [33] and corresponds to the data, that impurity content drastically decreases with IB size in solubilization and refolding.

### Validation of the impact of classical process parameters on IB QAs using *strain 2*

**Fig. 6:**
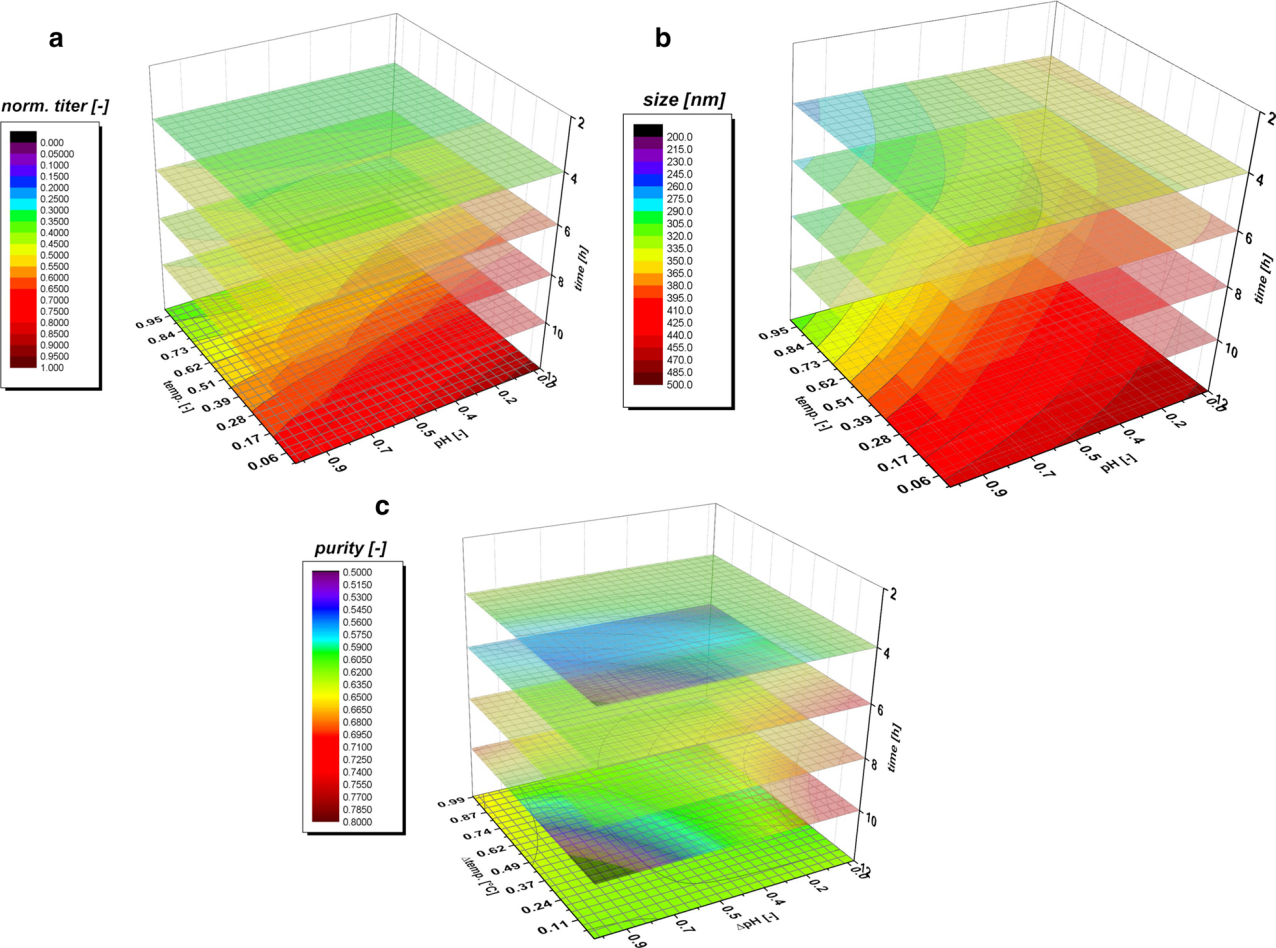
Data driven model for time dependent analysis of IB **a** titer, **b** size and **c** purity of *strain 2* using a reduced DoE design (Fig. 1b). Trends are given with differences of the lowest process value. Very similar behavior to *strain 1* can be found, showing highest purity, size and titer at values for low T and pH. Higher titers are produced using this strain resulting in boosted purities compared to *strain 1*. The analyzed size similar to *strain 1*

For application of the proposed QA dependence used for *strain 1*, a reduced design space (compare to Fig. 1b) for *strain 2* was applied and quality attributes were analyzed as described for *strain 1*. *Strain 2* also produces a toxic protein for the cells and is consequently expressed only as IBs but lacking the N-Pro fusion tag. As only four cultivations were performed, no statistical evaluation is used and fits were performed in order to have a reasonable model description and to reveal general trends during those cultivations. Estimation on standard deviations for the given QAs are already given in the previous section. In comparison to *strain 1* higher titers could be achieved during the cultivation. (Figure 6a—normalized to the highest achieved titer in these cultivations, given in 1 [−]). Time dependent analysis of the IB bead size is shown in Fig. 6b and reveals the same trend as already valid for titer and purity. Low pH and low temperatures lead to increased IB bead size in those cultivations. However, IB bead size is generally smaller in *strain 2*, when compared to *strain 1* respectively (N-Pro based protein, clearly visible by comparing Fig. 2b, d). The dimensionless value of purity is generally very high as well, exceeding values of 0.5 even after 4 h of induction, compare to Fig. 6c. In accordance to *strain 1* the highest titers and purities are found at a low pH and low temperatures.

Different IB bead size for a broad number of proteins was already presented in literature: A GFP model protein, expressing IBs as well as soluble protein [33] showed IB bead size of a maximum of 600 nm at extended induction times using mixed feed systems with glucose and lactose. Since, GFP also is expressed as soluble protein, only the ratio between IB and soluble protein is altered based on the feeding strategies. Producing a maximum size of 600 nm, the GFP-model protein forms an intermediate between the measured maximum of *strain 1* (N-Pro) and *strain 2* in this work. Other works report IB sizes between 502 nm for DnaK-IBs and 580 nm for ClpA-IBs [27] and approximately 600 nm for G-CSF IBs [28] and are in a reasonable range compared with our products in this work. IB bead size is strongly dependent on the produced product, on the polypeptide sequence and on hydrophobicity of the protein structure. IB QAs can accordingly be altered with the used classical process parameter T and pH, but morphological considerations have generally to be taken into account and can be product-based very different. Since IPTG concentration of 0.5 mM is high enough to induce all present cells, the secondary structure of the expressed proteins of *strain 2* has to inhere in higher density in their structure regarding the titers. Denser structures are much easier to be separated in centrifugation processes in the downstream, since the difference of the density compared to the host cell debris is far higher. This fact may also affect the purity and results in those high purity values for *strain 2.* Computer tomographic analysis of transmission electron microscopy (not shown) of *strain 1* reveal cavities within single inclusion bodies in the cell and may be the result for density variations of different IB products. Based on the findings for both strains in this study, time-resolved analytics of the IB QAs can be used to optimize the USP. Knowledge of titer as key performance indicator is important for determination of the harvest time point. The resulting IB bead size (and purity) is beneficial for planning of further necessary steps in the downstream for a given product.

### Impact of the physiological process parameter q_s,Glu_ on IB quality attributes of *strain 1* (N-Pro fused protein)

**Fig. 7:**
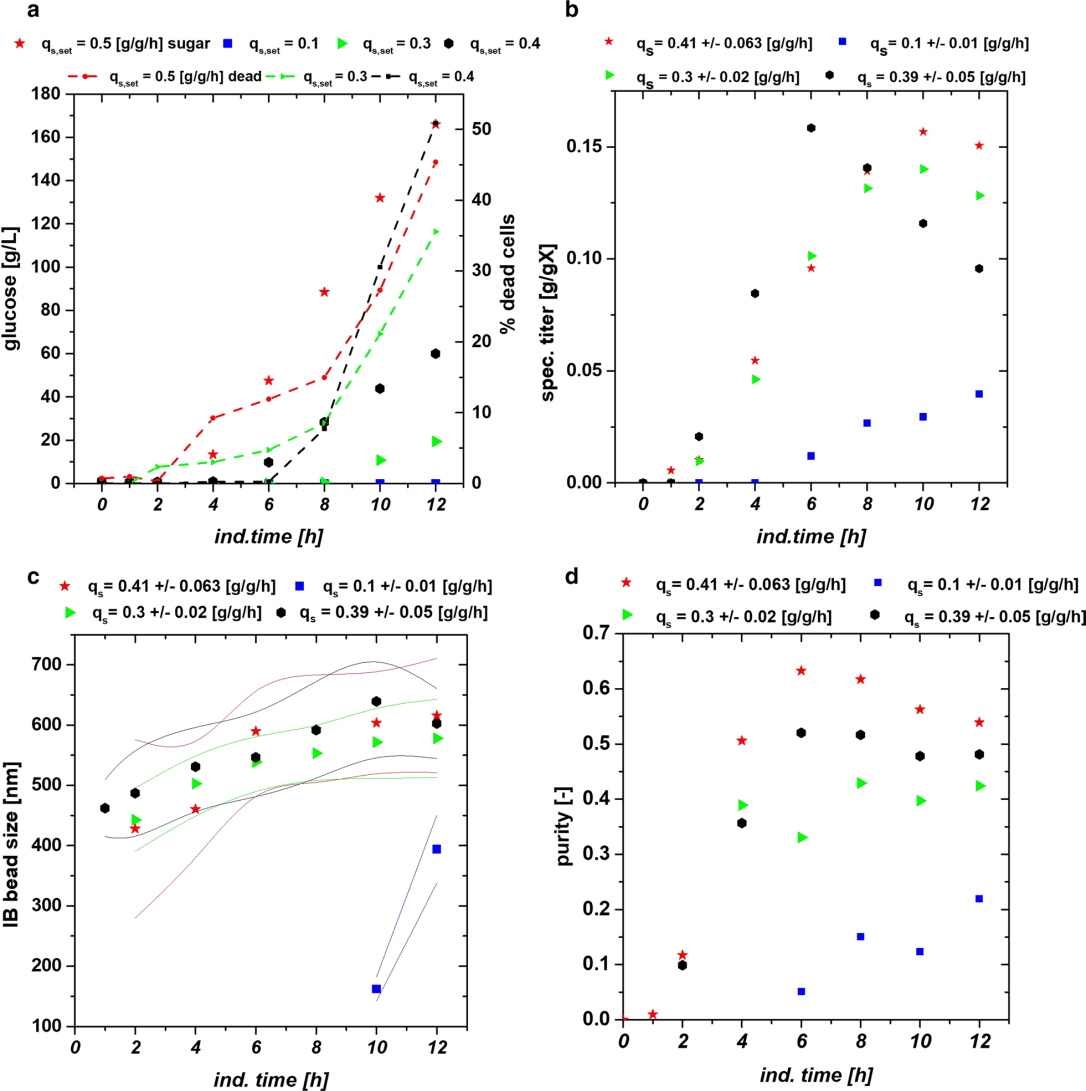
**a** Sugar-accumulation and cell death measured by FCM for three cultivations at different q_s,Glu_. Lowest q_s,Glu_ shows no cell lysis and accumulation; **b** specific titer of the recombinant protein fused to N-pro. Very high expression can be seen for the high q_s,Glu_ until 6 h with decreasing q_s,Glu_ also decreases product titer; **c** size of the IB beads. q_s,Glu_ = 0.41 g/g/h and q_s,Glu_ = 0.39 g/g/h are very similar. A very steep increase is followed by a steady state; q_s,Glu_ = 0.30 g/g/h shows increase over time, while size for q_s,Glu_ = 0.10 g/g/h is only detectable at 10 and 12 h of induction; **d** purity depicts clear dependence of all different q_s,Glu_ setpoints, increasing the IB purity with higher q_s,Glu_

Classical process parameters showed a high impact on IB properties during induction phase. The knowledge for optimized parameters for *strain 1*—was used for altering the physiological parameter q_s,Glu_. Temperature was decreased to 31.5 °C and pH was adapted to 6.7, while different setpoints for q_s,Glu_ were established during induction phase. Setpoints and real values for the q_s,Glu_ are given in Table 3. The induction characteristic of the four performed runs are given in Fig. 7a showing glucose accumulation and percentage of dead cells for the four performed cultivations. It was already investigated in literature that the correlation of growth rate and the production of recombinant protein resulted in a decrease in µ the more recombinant protein is produced [50]. This correlation could be clearly monitored in our study during induction phase when high titers of recombinant protein were produced. As consequence the growth rate (not shown) decreased, leading to sugar accumulations as the feed-rate over the whole induction phase was applied constantly [50]. Higher applied q_s,Glu_ resulted in early sugar accumulation and in increased number of dead cells in the cultivation and decreased the real q_s,Glu_ extensively even after some hours. After 12 h of induction 50% of the culture died at applied q_s,Glu_ of 0.4 and 0.5 g/g/h, while very low q_s,Glu_ showed neither cell death nor sugar accumulation. The time resolved titer measurements are given in Fig. 7b. Very high specific titers could be found at q_s,Glu-set_ = 0.5 g/g/h at 6 h of induction with highest volumetric productivities exceeding 1 g/L/h. However, the increased cell stress resulted in cell death and degradation of the product as could be seen in the decease of the titers at later time stages, respectively. After 12 h titers were almost identical irrespective of applied q_s,Glu_ for high setpoints (0.3–0.5 g/g/h). That indicated, time dependent analysis of QAs is therefore of utmost importance, especially at physiological process control. The peak value of the volumetric productivities (before degradation) showed a rising trend based on the mean q_s,Glu_ values which were applied (Additional file 4: Figure S4) and clearly indicated that the increased feeding rate is really beneficial for high productivity. The IB bead size given in Fig. 7c was generally very similar at q_s,Glu_ = 0.3–0.5 g/g/h applied values, with q_s,Glu_ = 0.3 g/g/h showing smaller diameters at later time stages. IB beads at q_s,Glu_ = 0.1 g/g/h were not detectable with SEM until 10 h of induction time. Low q_s,Glu_ yielded very small IB sizes and low titers in Fig. 7b as only low energy is available for production of the recombinant protein. A steep increase in the beginning of the induction time was generally accompanied by leveling off in diameter at later stages. Trends for IB purity are given in Fig. 7d. Higher q_s,Glu_ values were beneficial for protein purity, which were in reasonable accordance with trends for titer and size already seen in the previous chapter.

**Table 3:** Applied q_s,Glu_ vs. real q_s,Glu_ values after reverse analysis of the cultivation data

Run	q_s,Glu set_	q_s,Glu real_
1	0.1	0.1 ± 0.01
2	0.25	0.3 ± 0.02
3	0.4	0.39 ± 0.05
4	0.5	0.41 ± 0.063

Sugar accumulation and cell death at higher applied values result in higher standard deviations

Based on these findings improved control strategies for IB production could be established in further development steps using the optimized process parameters for the two used strains in combination with physiological process control (time dependent adaption of the specific substrate uptake rate) during the induction phase.

## Conclusions

IB quality attributes were analyzed in respect of changes in classical process parameters pH and T in the induction phase. Pronounced changes in QAs could be found in the analysis of IB titer, IB bead size and IB purity. Optimized process conditions for *strain 1* were found to be at pH 6.7 and 31.5 °C during induction in respect of the produced maximum IB titer. These findings were checked using a second industrial relevant strain, revealing that low temperatures and low pH is highly beneficial for production of IBs. Therefore, we would like to hypothesize that yields of exclusively IB based products can be improved by applying low temperatures and a relatively low pH value during the induction phase as analyzed in this study for two very different products. Despite of this platform knowledge, absolute values for size, titer and purity were strongly product dependent and exhibit very different values for every produced product.

The sweet spot conditions (pH 6.7, T = 31.5 °C) for *strain 1* were used to show the impact of physiological control onto IB quality attributes. The four performed cultivations exhibited different specific substrate uptake rates (q_s,Glu_) and revealed high impact on analyzed IB QAs. High constantly applied q_s,Glu_ boosted titer, bead size and purity very early in the induction phase, but resulted generally in high glucose accumulation and cell death, while low q_s,Glu_ did not stress the cells, but lead to very low production of IBs. Physiological control based on these findings may be highly industrially relevant in order to find IB parameters with high productivity, but also low contamination of host cell proteins and DNA.

We would also like to highlight that time dependent monitoring of the here defined IB-QAs can be used as a tool to optimize process parameters such as pH, temperature and (q_s,Glu_). By improving the upstream conditions, we aim to trigger robust downstream procedures, increasing the overall time/space yield of IB-processes.

## Additional files

**Additional file 1: Figure S1.** Analysis of the first center point run representing IB purity. Buffer washed samples showed generally higher purity. Differences in size and titer are within the given standard deviation.

**Additional file 2: Figure S2.** a) Electropherogram for two different timepoints during a cultivation (4 h and 12 h). A clear visibility of impurity pattern near the protein of interest (high peak after 28 kDa) is given; b) SDS-Page for visualization of DNA related impurities. No distinct differentiation can be made between DNase treatment and virgin sample.

**Additional file 3: Figure S3.** a) Mean value for size and deviations of the three individual center point runs. Error stays constant; b) purity-based analysis, with decreasing error over time; c) titer-based analysis. Error decreases drastically in later time stages (range of constant titer or even proteolytic degradation).

**Additional file 4: Figure S4.** q_s,real_ with standard deviation based on the reverse analysis. The higher the q_s_ the higher is the error, due to onset of degradation and sugar accumulation in the broth. A rising trend can be dedicated from these measurements.

## Abbreviations

ACN: acetonitrile
AFM: atomic force microscopy
DCW: dry cell weight
dO_2_: dissolved oxygen
DoE: design of experiments
DSP: downstream processing
FCM: flow cytometry
GFP: green fluorescent protein
IB: inclusion body
IPTG: isopropyl β-d-1 thiogalactopyranoside
IR: infrared
MQ: ultrapure water
QA: quality attribute
q_s,Glu_ (g/g/h): specific substrate uptake rate (glucose)
r_p_ (g/L/h): volumetric productivity
SEM: scanning electron microscopy
TCA: tricarboxylic acid (cycle)
TEM: transmission electron microscopy
USP: upstream processing
UV: ultraviolet
