# Supplementary figures and images for: Custom made inclusion bodies: impact of classical process parameters and physiological parameters on inclusion body quality attributes
Source: Microb Cell Fact. 2018 Sep 20;17:148. doi: 10.1186/s12934-018-0997-5 (PMC6148765; doi:10.1186/s12934-018-0997-5)

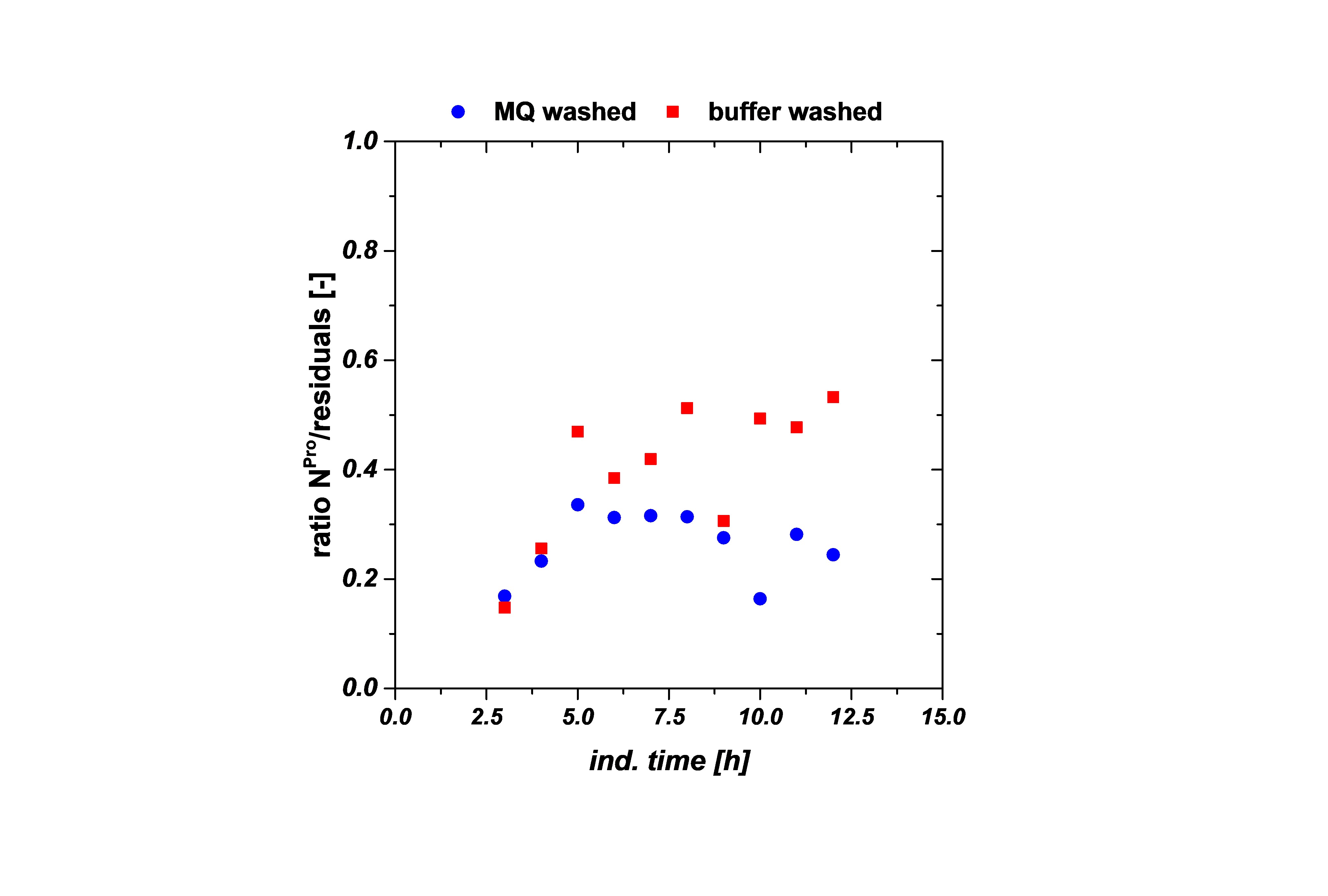

Supplement: Supplementary file 1 — Additional file 1: Figure S1. Analysis of the first center point run representing IB purity. Buffer washed samples showed generally higher purity. Differences in size and titer are within the given standard deviation. [file 12934_2018_997_MOESM1_ESM.jpg]

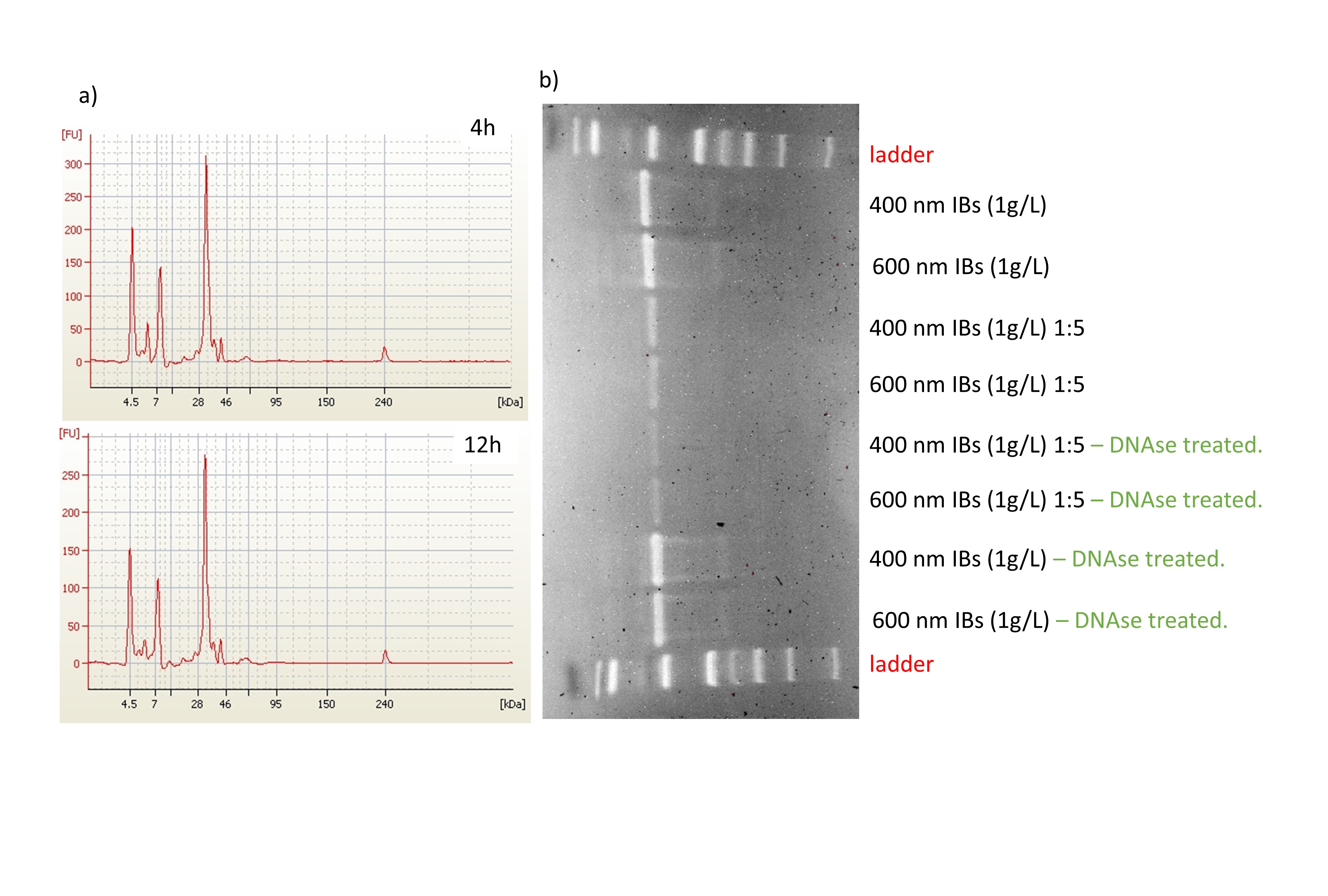

Supplement: Supplementary file 2 — Additional file 2: Figure S2. a) Electropherogram for two different timepoints during a cultivation (4 h and 12 h). A clear visibility of impurity pattern near the protein of interest (high peak after 28 kDa) is given; b) SDS-Page for visualization of DNA related impurities. No distinct differentiation can be made between DNase treatment and virgin sample. [file 12934_2018_997_MOESM2_ESM.jpg]

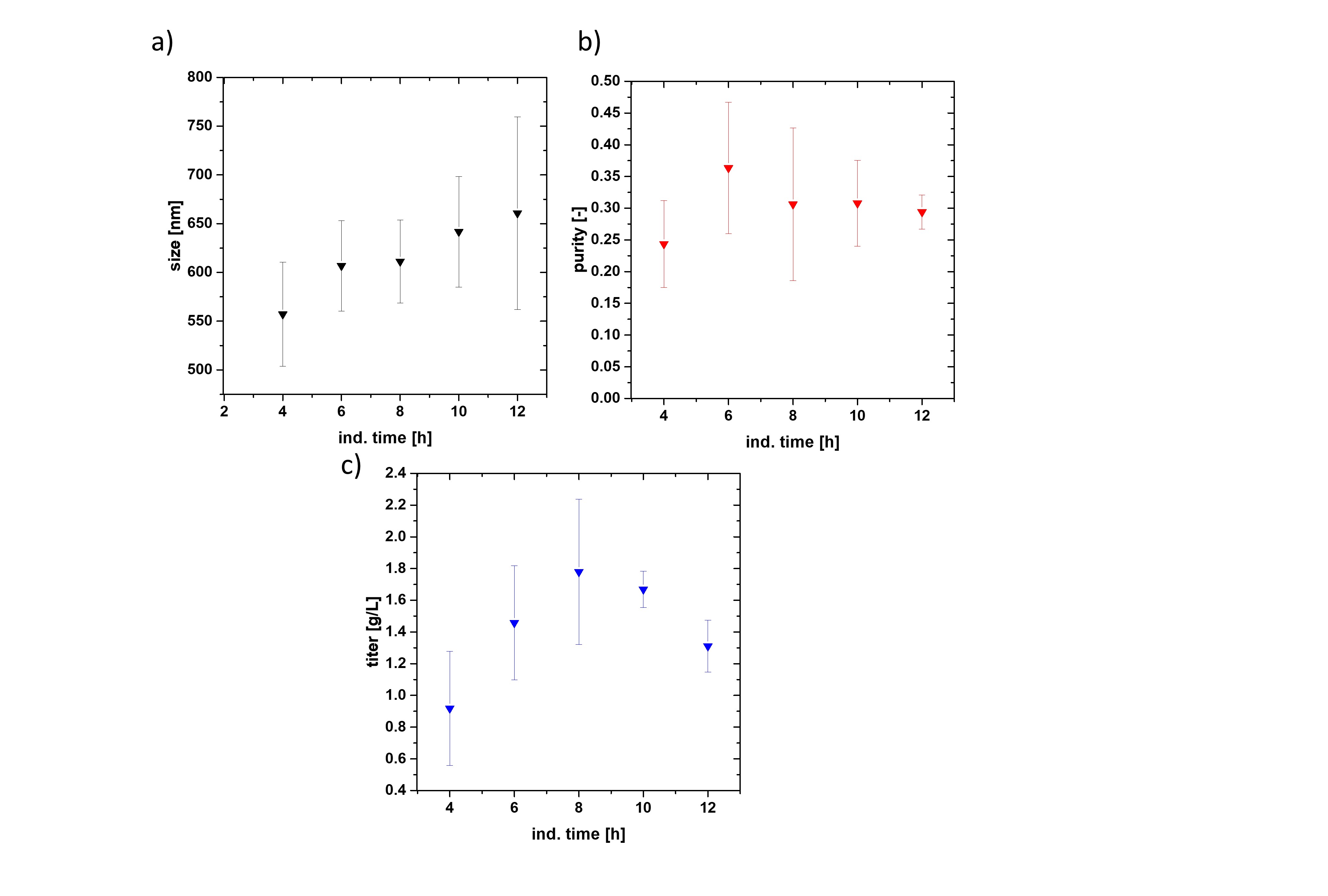

Supplement: Supplementary file 3 — Additional file 3: Figure S3. a) Mean value for size and deviations of the three individual center point runs. Error stays constant; b) purity-based analysis, with decreasing error over time; c) titer-based analysis. Error decreases drastically in later time stages (range of constant titer or even proteolytic degradation). [file 12934_2018_997_MOESM3_ESM.jpg]

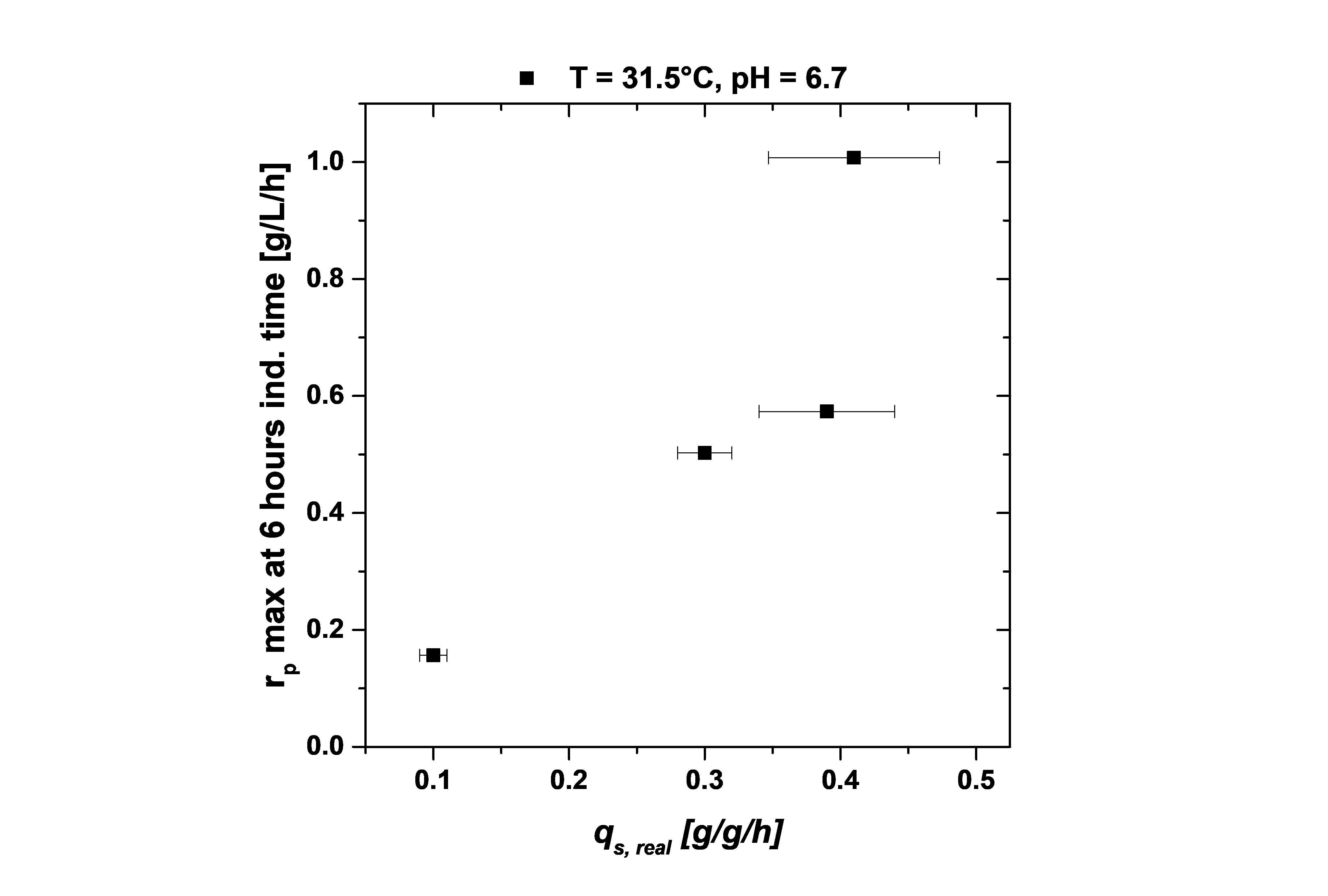

Supplement: Supplementary file 4 — Additional file 4: Figure S4. qs,real with standard deviation based on the reverse analysis. The higher the qs the higher is the error, due to onset of degradation and sugar accumulation in the broth. A rising trend can be dedicated from these measurements. [file 12934_2018_997_MOESM4_ESM.jpg]
